# The role of self-care and self-compassion in networks of resilience and stress among healthcare professionals

**DOI:** 10.1038/s41598-025-01111-1

**Published:** 2025-05-27

**Authors:** Carolina Pank, Lisa von Boros, Klaus Lieb, Nina Dalkner, Sebastian Egger-Lampl, Dirk Lehr, Sarah K. Schäfer, Oliver Tüscher, Michèle Wessa

**Affiliations:** 1 Leibniz Institute for Resilience Research, Mainz, Germany; 2 Department of Psychiatry and Psychotherapy, University Medical Center of Johannes Gutenberg University Mainz, Mainz, Germany; 3 Department of Psychiatry and Psychotherapeutic Medicine, Medical University Graz, Graz, Austria; 4 Mindconsole GmbH, Graz, Austria; 5 Institute for Sustainability Education and Psychology, Department of Health Psychology and Applied Biological Psychology, Leuphana University, Lüneburg, Germany; 6 Department of Clinical Psychology, Psychotherapy and Psychodiagnostics, Technical University Braunschweig, Braunschweig, Germany; 7 Department of Psychiatry, Psychotherapy and Psychosomatic Medicine, Martin-Luther University Halle-Wittenberg, Halle, Germany; 8 Central Institute of Mental Health, Mannheim, Germany; 9 DKFZ Hector Cancer Institute at the University Medical Center Mannheim, Heidelberg, Germany; 10 German Cancer Research Center (DKFZ) Heidelberg, Division C160, Heidelberg, Germany

**Keywords:** Network models, Healthcare professionals, Resilience, Self-Care, Self-Compassion, Burnout, Psychology, Human behaviour

## Abstract

Healthcare professionals (HCPs) are essential for maintaining our healthcare system but are at risk for developing mental health issues due to chronic occupational stress. This can lead to a vicious cycle with extended sick leave, increased workloads for colleagues, and strain on the healthcare system. Therefore, preventive interventions aiming at enhancing resilience - the maintenance of mental health despite stress - are essential. Yet, identifying the most impactful resilience factors has been challenging. To explore the relationships between resilience factors, stress, mental health, and work-related outcomes, we conducted regularized partial correlation network analyses focusing on self-care and self-compassion. Cross-sectional data from HCPs in Germany were collected in April 2023. Analyses of 212 HCPs (age 41.63 [21–68] years; 81.60% women) revealed self-compassion as the most important factor across all networks, while the importance of self-care showed through individual connections to crucial factors like mental health problems and work-life balance. Work engagement, contrary to burnout, was closely interrelated with resilience factors. In conclusion, despite accounting for established evidence-based resilience factors, self-compassion and self-care seem crucial in the context of stress and mental health in HCPs. More research is needed to validate the causal importance of self-care and self-compassion.

## Introduction

Healthcare professionals (HCPs) are often exposed to a substantial amount of work-related stress, including time pressure and workload, having multiple roles, dealing with death, and high levels of responsibility in difficult medical decisions^1–3^. This can lead to chronic distress and role dissatisfaction, and is reflected in a high prevalence of stress-related mental health problems such as burnout, anxiety, or depressive symptoms^3–5^. Beyond the ethical need to support HCPs as individuals, this professional group is critical for maintaining efficiency and quality in healthcare systems. Sustaining the mental health of HCPs is crucial, as studies highlight that those who report higher levels of fatigue and dissatisfaction are also more prone to medical errors^6^. Moreover, increased patient-to-nurse ratios are associated with an increased likelihood of burnout and job dissatisfaction, greater patient mortality, and higher failure-to-rescue rates^7^. Conversely, favorable work environments for nurses, such as having adequate staff, good administrative support for nursing care, and good relations between doctors and nurses, were linked to patient satisfaction with care as well as reduced risk of turnover and job burnout for nurses^8^. Therefore, understanding and fostering resilience among HCPs is not only a matter of their individual well-being, but also a critical component of maintaining a safe and effective healthcare system.

With the concept and understanding of resilience significantly changing over time, we follow the recommendations to define resilience using an outcome-oriented approach^9^. Therefore, resilience can be defined as the maintenance or quick recovery of mental health during or after stress exposure^10,11^. Resilience is understood here as a dynamic process in which resilience factors, such as e.g. optimism and self-efficacy, serve like a buffer against potential negative effects of stress^12^. However, psychological constructs, proposed as resilience factors, conceptually overlap, complicating the investigation of their differential contribution to resilience, as well as their interplay and their combined effects^12,13^. Therefore, more holistic models capturing the complexity of the concept are needed.

Along with the movement of understanding mental disorders as complex networks of interacting symptoms rather than mere manifestations of a latent disorder^14,15^, some authors proposed applying such a network approach to resilience research^11^. Network modeling in psychological research offers a relatively new and dynamic approach that reflects the complexity of psychological phenomena, moving beyond the oversimplified notion that symptoms or traits share one underlying cause^16^. The most common method is to estimate networks of partial correlation coefficients, called Gaussian graphical models^17^. In these networks, psychological constructs are represented as nodes, with edges indicating positive or negative associations that control for all other variables. Visualizing the relationships allows for a nuanced understanding of psychological processes and captures complex interactions between variables and their reciprocal, direct, and indirect influence. Recent studies have already applied network analysis to resilience research in various contexts, such as stress, adversity, and burnout^12,13,18–21^. These studies are mostly limited to cross-sectional data, which restricts conclusions about predictive values and leaves causal directions unclear. However, this approach excels in identifying the structure of resilience networks, allowing for the identification of vulnerability- and protective factors within a network. Network studies contribute to uncovering key nodes and pathways and highlight the importance of certain factors in promoting resilience.

With this dynamic approach in mind, it is essential to examine which resilience factors may be important for HCPs. This can contribute to identifying central factors and important connections, guiding the development of preventive resilience interventions. An extensive review on resilience interventions for HCPs examined the best-evidenced, modifiable resilience factors across various populations^22^. Based on the summary of several systematic reviews, the strongest evidence for factors associated with resilience emerged for active coping (e.g. problem solving, planning), self-efficacy, optimism, social support, cognitive flexibility (e.g. positive reappraisal, acceptance of negative situations and emotions), and religiosity/spirituality^22^. However, these factors often only explain a small amount of variance in resilient outcomes^23^. Therefore, it is important to zoom in and take additional factors into consideration that may be of interest for the resilience of HCPs.

Self-care and self-compassion have been considered to potentially play an important role in resilience in the context of helping professions^24–26^. Self-compassion describes a healthy, emotionally positive, and compassionate relationship to oneself, comprising the three components self-kindness, common humanity, and mindfulness^27^. The development of a compassionate, nonjudgmental, and sensitive approach to oneself has been linked to a compassionate approach towards others in HCPs, thus potentially hindering compassion fatigue, which, in turn, is linked to burnout^28–30^. In the literature, momentary self-compassion has been associated with better emotional responses to daily hassles, such as reduced negative affect and preserved positive affect^31^. Moreover, studies on high-risk groups have shown that self-compassion serves as a moderator in the relationship between self-criticism and depressive symptoms^25^. Also work-engagement, described as the positive antithesis of burnout^32^, has been associated with self-compassion, as self-compassionate HCPs show more positive work engagement, feel less exhausted by work demands, and are more satisfied with their professional lives^33^.

Self-care, though defined in various ways in the literature, can be understood as the active engagement in behaviors and practices that support physical, emotional, and social well-being^34^. A growing body of literature emphasizes the importance of HCPs engaging in self-care as a preventative approach to hamper negative mental health outcomes for themselves and their clients. Moreover, self-care is thought to prevent the downward spiral of stress, burnout, and professional impairment, and to promote an upward spiral of well-being instead^35^. Self-compassion and self-care can be understood as principles that support and complement each other. While self-compassion represents the part of the caring attitude, of “being good to oneself”, self-care represents the corresponding active action, i.e., “doing something good for oneself”^36^. Some research suggests that self-care is fueled by self-compassion^37^, which has been identified as a predictor of self-care^38^, associated with health-promoting behavior^39,40^, or shown to foster more intentional self-care practices in intervention studies^41^.

To sum up, self-compassion and self-care have already been investigated in relation to work-related factors such as burnout, work engagement, and work-life balance among HCPs. However, to the best of our knowledge, no study has yet examined their shared influence on all these factors together in one model, while simultaneously accounting for the influence of other well-established resilience factors. Investigating these factors altogether as a network allows us to capture their combined and unique contributions within a single comprehensive model. By including both self-compassion and self-care alongside established resilience factors, we aim to contribute to a better understanding of how these protective factors interact, offering more nuanced insights into resilience-building mechanisms among HCPs.

Hence, we first want to examine the interrelations of self-compassion and self-care with other evidence-based resilience factors among HCPs to uncover how these resilience factors dynamically interact to contribute to overall resilience (research objective (RO) 1). In doing so, we are accounting for resilience as the overall product of the unique interactions of resilience factors, which in turn are characterized as more stable traits. However, we do not claim to paint a complete picture of resilience, but rather want to explore how the interactions of a selected set of resilience factors unfold when taking self-care and self-compassion into account. Therefore, when including self-care and self-compassion in the model with resilience factors, we refer to them as resilience-related factors, as they have not yet been widely recognized as core resilience factors in the literature. Second, since resilience is defined in response to stress, we want to investigate the relationships of the resilience-related factors with micro-stressors and mental health to examine the incremental correlation of self-compassion and self-care besides evidence-based resilience-factors (RO2). Third and lastly, we want to examine the relationship between work-related factors, self-compassion, and self-care, and resilience factors (RO3). Thereby, we aim to gain insights about the interrelations of resilience-related factors with work-related outcomes. Additionally, we examine in what way self-compassion and self-care may have additional value when considered alongside well-established resilience factors in the same model.

## Methods

The data analyzed in the present study stem from a randomized-controlled trial (RCT) evaluating the effectiveness of an online resilience intervention for HCPs, preregistered on ClinicalTrials.gov (Identifier: NCT05812716). The present paper presents a secondary analysis of the baseline data from the RCT, which is why its specific research question and methodology were not part of the preregistration. Accordingly, no a priori sample size calculation was conducted for the current analyses. The RCT was conducted from June 2023 to July 2024, with the baseline assessment taking place from 20/06/23 to 04/07/23, prior to randomization to receive the online resilience intervention.

Participants for this study were partly recruited from the pool of the *Gutenberg Brain Study* (https://lir-mainz.de/en/gutenberg-brain-study). This is a large, socioeconomically diverse cohort of healthy individuals living in Mainz, Germany, who consented to be contacted for future research projects. Additionally, participants were recruited via hospitals, professional associations, training institutions (e.g., Universities with nursing degree programs), and counseling and care facilities, where the resilience training for HCPs was advertised.

Eligible participants were ≥ 18 years old, fluent in German, and working at least 10 h per week in a healthcare profession (e.g., doctors, nurses, psychotherapists, speech therapists, physiotherapists, dentists, medical-technical assistants, geriatric nurses). They also had to meet organizational requirements such as access to a computer with internet. Participants were excluded when they reported high levels of mental burden (e.g., reporting suicidal ideation, receiving psychotherapy, or psychiatric treatment).

Participation in the baseline assessment was compensated with 10€. Data were collected online via SoSci Survey^42^. A total of 244 HCPs participated in the baseline assessment. For the present analyses, only participants who completed the full baseline survey and self-rated their data as usable were included (*n* = 223). Further, following quality control recommendations^43^, participants with a relative speed index above 2.0 (i.e., completing the survey in less than half the median completion time) were excluded, yielding a final sample of 212 HCPs.

All participants provided informed consent. The study was conducted in accordance with the declaration of Helsinki and its latest revisions, and approved by the ethics committee of the Rhineland-Palatinate Medical Association (Ethics approval ID: 2022–16888).

### Measures

#### Resilience-related factors

*Optimism* as a personality trait describes the generalized expectation that positive outcomes will occur across various aspects of life, reflecting a broad sense of confidence in favorable future events^44^. Optimism was measured using the German optimism-pessimism Scale (SOP-2)^44^, a two-item short scale to assess dispositional optimism and pessimism on a 7-point Likert scale. The SOP-2 has demonstrated adequate to good reliability and validity, as shown in a validation study based on two samples^45^. However, literature suggests optimism and pessimism are not polar opposites from a single dimension, but rather distinct constructs that have demonstrated independent relationships with aspects of health and well-being^46^.Therefore, only the single optimism item was included in the analyses, with higher values indicating more optimism.

*Problem-focused coping* was assessed with the respective subscale of the German brief version^47^ of the Coping Orientation to Problems Experienced Inventory (Brief-COPE)^48^. The Brief-COPE is a 28-item questionnaire designed to assess coping behavior (i.e., the self-perceived general tendency to cope in a specific way) on a 4-point Likert scale. However, in a German validation study, it was recommended for research purposes to analyze data only on a second factor level, since factors on the first level have lower reliability^49^. Therefore, we followed the division of the Brief-COPE into three factors: (1) problem-focused coping, (2) emotion-focused coping, and (3) avoidant coping^50^, and used the problem-focused coping subscale of the three-factor model for our analyses. The problem-focused coping subscale includes four single coping strategies (i.e., active coping, the use of informational support, planning, positive reframing), with higher scores indicating a more practical approach to problem solving. The problem-focused subscale comprises 8 items that are used for calculating the mean score. The subscale showed an acceptable internal consistency in our sample, with Cronbach’s alpha (α) = 0.75 and McDonald’s omega (ω) = 0.78.

General *self-efficacy* expectations reflect the assessment of one’s own ability to successfully plan and execute actions to achieve desired goals^51^. Self-efficacy beliefs were assessed using the 3-item German short scale for measuring general self-efficacy beliefs (ASKU), developed and validated in the scope of three empirical studies^51^. This scale was chosen for its economy, providing a reliable and valid measurement of the construct while demonstrating good psychometric properties. Each item was rated on a 5-point Likert scale, with higher mean scores indicating stronger self-efficacy beliefs. The scale showed good internal consistency (α = 0.82; ω = 0.83).

*Social support* is a psychosocial resource available through interpersonal contacts and social networks, encompassing structural aspects like the size and type of social networks as well as the frequency of interactions, and functional aspects related to the experience or expectation of obtaining support from relationships^52^. Social support was measured using the German version^52^ of the 3-item Osloer Social Support Scale (OSSS-3)^53^. The OSSS-3 was chosen for its brief and economic approach, offering a concise measure of social support while maintaining strong psychometric properties as shown in the German validation study^52^. While a distinction is often made between received and perceived social support, the scale used here is based on a uniform concept. The scale consists of only three items that ask for the number of close confidants, the sense of concern from other people, and the relationship with neighbors with a focus on the accessibility of practical help. One item is rated on a 4-point Likert scale, while the remaining two items are assessed on a 5-point Likert scale. The sum score of all three items was built, with larger scores indicating higher levels of social support. The internal consistency was acceptable and similar to the internal consistency reported in the validation study^52^ (α = 0.65; ω = 0.7).

*Self-compassion* was measured using the German version^54^ of the Self-Compassion Scale (SCS-D)^55^. The scale consists of 26 items that are rated on a 5-point Likert scale and has been validated with two German-speaking samples^54^, showing good psychometric properties. It comprises the subscales self-kindness, common humanity, mindfulness, reduced self-judgment, isolation, and overidentification. While it has been discussed whether separate scores representing compassionate versus uncompassionate self-responding should be used, Neff and colleagues support the use of the total score to represent overall self-compassion^56^. Therefore, we proceeded building the mean score over all items with higher scores indicating higher levels of self-compassion. The scale showed an excellent internal consistency (α = 0.92; ω = 0.93).

*Self-care* was measured using the Hamburg Self-Care Questionnaire (HSF)^34^, a multidimensional, German-language instrument with good psychometric properties. The scale was chosen for its theoretically grounded and multidimensional approach to assessing self-care and its development and validation in a German-speaking sample. The scale consists of 12 items divided into the subscales pacing and positive experience. Pacing refers to a mindful approach to oneself and personal boundaries, while the latter subscale focuses on the acceptance and enjoyment of positive experiences and behavior. The items are rated on a 5-point Likert scale and built to a total score with higher scores indicating a higher level of self-care. The internal consistency was excellent (α = 0.91; ω = 0.92).

#### Stress and mental health

*Stress* exposure was assessed using the Mainz Inventory for Micro Stressors (MIMIS)^57^. The MIMIS assesses the frequency of occurrence of 58 micro stressors, i.e., daily hassles, within the past week. After rating how often a stressor – such as losing or misplacing objects or encountering a negative event in the media - occurred within the last 7 days (between zero and seven times), people rate their subjective burden caused by this stressor. As people are susceptible to stress in different ways, we decided to build sum scores solely based on the frequency of the occurrence of specific stressors. We did not take into account subjective burden, as such ratings are at risk of being confounded with overall mental distress^58^. The MIMIS was chosen for its broad coverage of relevant stressor domains, it’s clear distinction between frequency and subjective burden, and its ecological validity in capturing the diversity of daily stress experiences with strong psychometric quality. The MIMIS had overall good internal consistency (α = 0.89; ω = 0.89).

*Mental health problems* were assessed using the German version^59^ of the General Health Questionnaire (GHQ-12)^60^, a widely used screening instrument for psychological distress that has been validated in several populations^61,62^. The GHQ-12 was chosen for its brevity, broad applicability across clinical and non-clinical settings, and strong psychometric properties. The 12-item scale assesses mental health domains such as sleep, mood, symptoms of depression, and anxiety in the last few weeks. Each item had to be rated on a 4-point Likert scale and was summed up to an overall sum score with higher scores indicating more mental health problems, i.e., worse mental health. The questionnaire demonstrated good internal consistency (α = 0.84; ω = 0.84).

#### Work-related outcomes

*Burnout* was originally defined as a response to chronic stress in emotionally demanding client work, marked by emotional exhaustion, cynicism, and negative self-evaluation. It reflects depleted emotional resources and often leads to detachment and dissatisfaction with one’s professional role^63^. Burnout was assessed using the German version^64^ of the 25-item Maslach Burnout Inventory (MBI-D)^65^, one of the most widely used and well-validated instruments for measuring work-related burnout. The MBI-D was chosen due to its strong psychometric properties, its multidimensional conceptualization of burnout, and its wide applicability in occupational health research, particularly in healthcare settings. Each item was rated on a 7-point Likert scale with the additional option to indicate “not applicable”. The MBI-D assesses the burnout dimensions depersonalization, emotional exhaustion, and personal accomplishment, with higher scores indicating higher levels of the respective domain. While higher scores for emotional exhaustion and depersonalization indicate more burnout, the opposite is true for personal accomplishment, thus less of the latter leading to more burnout. In line with the manual recommendations and extensive literature, sum scores of the domains were calculated separately, not building an overall burnout score^65^. The subscales of the MBI-D showed good internal consistencies for emotional exhaustion (α = 0.89; ω = 0.89) and depersonalization (α = 0.82; ω = 0.82), and acceptable internal consistency for personal accomplishment (α = 0.74; ω = 0.78).

*Work-engagement* is defined as a positive work-related state of fulfillment that is characterized by vigor, dedication, and absorption. It was assessed using the German version^66^ of the Utrecht Work Engagement Scale (UWES-9)^67^, a widely used and well-validated short questionnaire. The 9-item scale comprises three subscales assessing the components vigor (vitality), dedication (commitment), and absorption, which can be summarized into a general index of work engagement, with higher mean scores indicating greater work engagement. The UWES-9 was chosen due to its brevity, excellent psychometric properties, and its applicability across occupational groups, allowing for reliable assessments. Items were rated on a 7-point Likert scale and showed excellent internal consistency in our sample (α = 0.94; ω = 0.98).

*Work-life balance* encompasses the attitude towards one’s life situation, particularly the compatibility of various life domains, roles, and goals. A good work-life balance therefore means that the actual organization is in harmony with the desired balance. It was measured using the 5-item Trier Short Scale for Measuring Work-Life Balance^68^. This brief, well-validated German instrument has been widely used in research across different occupational settings and was chosen for its strong psychometric properties. Each item is rated on a 6-point Likert scale, and higher mean scores indicate a better balance between work and life. The scale demonstrated good internal consistency (α = 0.85; ω = 0.86).

### Statistical analyses

All statistical analyses were performed with R version 4.2.3 (2023-03-15).

#### Network estimation

For network analyses, the R packages *qgraph*^69^, *mgm*^70^, and *bootnet*^71^ were used. In psychological networks, variables are represented as nodes and their connections are called edges. In partial correlational network models, edges are controlled for the relationship of other variables included in the model. With our relatively small sample size, we followed the recommendations to choose regularized estimators to discover a network structure that resembles a true network and discovers the strongest edges^72^. Therefore, partial correlational networks were calculated, using the graphical least absolute shrinkage and selection operator (*glasso*) as regularization technique. The least absolute shrinkage and selection operator (*LASSO*) applies a regularization penalty by constraining the total sum of absolute parameter values^73^. As a result, LASSO produces sparse network models in which relatively few edges are retained, making the model structure easier to interpret^73^. The degree of penalization, and thus the sparsity of the network, is controlled through a tuning parameter^72^. To select this tuning parameter, we used the Extended Bayesian Information Criterion (EBIC)^74^, which returns a set of network models and chooses the one with the best fit^72^. This approach was chosen as LASSO regularization with EBIC model selection provides high specificity^17^. Furthermore, the *EBICglasso* estimator has been shown to be robust when interpreting centrality indices for small samples^72^.

The EBIC improves upon the traditional Bayesian Information Criterion (BIC) by introducing a hyperparameter γ that adds an extra penalty for model complexity^72^. When γ is set to 0, EBIC reduces to the ordinary BIC without any additional penalization. Higher γ values increase the penalty, making sparser models more likely to be selected, where only the most important associations remain in the network^75^. While γ = 0.5 is known to perform well^74,76^, the optimal value depends on the trade-off between sensitivity and specificity: lower γ values improve the chance of detecting true edges (higher positive selection rate), whereas higher values reduce false positives (lower false discovery rate)^74^. Since our aim was rather exploratory, to identify strong edges in a small sample with skewed ordered categorical data, we opted for a more sensitive edge selection criterion that does not penalize model complexity too strongly. Therefore, the hyperparameter was set to γ = 0.25, allowing the model to be more flexible and detect more ‘true’ edges. We are thus following recent literature^12,77^, enforcing higher sensitivity compared to the commonly used value of γ = 0.5. However, to ensure the robustness of our findings, sensitivity analyses were conducted by running additional models with the commonly recommended value of γ = 0.5^76^. A threshold was not employed for a more sensitive approach.

To identify the most influential factors, i.e., the factors with the strongest overall relationships in the network, *strength centrality* was used, which measures the sum of absolute edge weights for each node. Strength as a centrality measure was found to show the highest correlations between the true and estimated network structures, while closeness performed worse and betweenness was recommended not to be used anymore^72^. To assess the network accuracy, first, the edge-weight accuracy was estimated by drawing 2,500 bootstrapped 95% confidence intervals using non-parametric bootstrapping. Second, to investigate the stability of the centrality indices, case-dropping subset bootstrapping was used, to examine the correlation stability coefficient (*CS*-coefficient, setting: 0.70)^73^. The CS-coefficient with a setting of correlation (cor) cor = 0.70 indicates the maximum percentage of cases that can be removed while still ensuring, with 95% confidence, that the correlation between the centrality indices of the original network and those of the subset-based networks remains at least 0.7^73^. The CS-coefficient should ideally be above 0.5 and not below 0.25 to interpret centrality differences.

First, we estimated a network only including resilience-related factors to explore interrelationships among these variables (*Network on resilience-related factors*). Second, we included daily hassles and mental health problems to the network on resilience-related factors (*Network on resilience-related factors and indicators of stress*). In the last step we replaced the stress-related variables with work-related factors, including the three distinct facets of burnout, work engagement, and work-life balance while still retaining the resilience-related factors in the network (*Network on resilience-related factors and work-related outcomes*).

## Results

### Sample characteristics

The final sample comprised 212 HCPs, the majority of whom self-identified as women. The most common professional groups were speech therapists, nurses, and physiotherapists. A detailed overview of the sociodemographic and occupational characteristics is provided in Table 1.

**Table 1:**
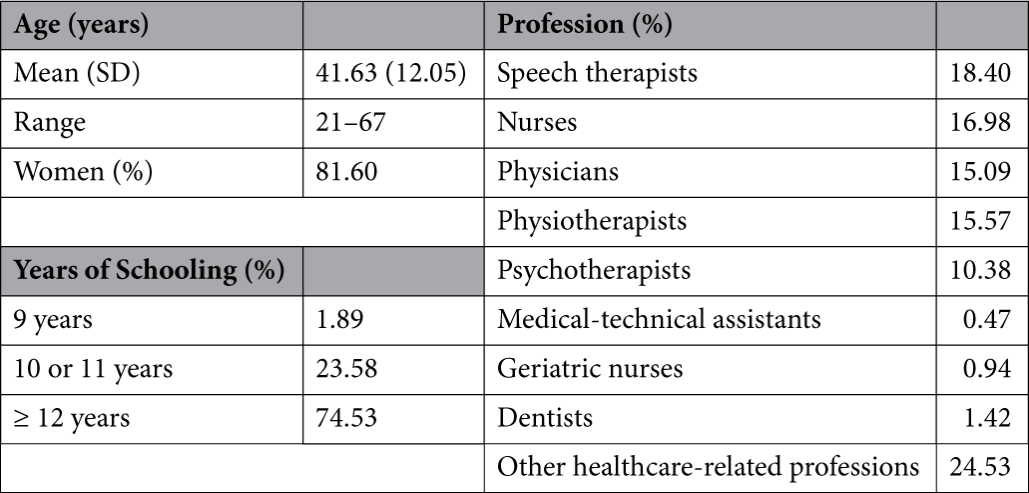
Sociodemographic and occupational data of HCPs. *N* = 212; SD = standard deviation.

As this study is part of a larger RCT for which completion of the baseline assessment was mandatory, the data were complete with no missing data on single items or dropout.

### Descriptive statistics

Table 2 presents the means, standard deviations, and bivariate correlations, including their confidence intervals for all variables examined in the network models.

**Table 2:** Means and standard deviations of scales, and bivariate correlations with confidence intervals between the scales.

Variable		1	2	3	4	5	6	7	8	9	10	11	12	
1. Optimism		5.02 (1.37)												
2. Self-compassion		0.48**	3.22 (0.62)											
		[0.37, 0.58]												
3. Social support		0.32**	0.22**	10.25 (2.06)										
		[0.19, 0.44]	[0.09, 0.34]											
4. Self-efficacy		0.40**	0.45**	0.25**	4.06 (0.56)									
		[0.28, 0.51]	[0.34, 0.55]	[0.12, 0.37]										
5. Problem-focused coping		0.35**	0.45**	0.37**	0.36**	2.84 (0.48)								
		[0.23, 0.46]	[0.34, 0.55]	[0.25, 0.48]	[0.24, 0.47]									
6. Self-care		0.29**	0.49**	0.35**	0.31**	0.34**	3.77 (0.70)							
		[0.16, 0.41]	[0.38, 0.59]	[0.23, 0.47]	[0.19, 0.43]	[0.22, 0.45]								
7. Micro-stressors		− 0.15*	− 0.25**	− 0.22**	0.01	− 0.13	− 0.35**	79.09 (35.70)						
		[-0.27, − 0.01]	[-0.38, − 0.12]	[-0.34, − 0.08]	[-0.12, 0.15]	[-0.26, 0.00]	[-0.47, − 0.23]							
8. Mental health problems		− 0.33**	− 0.49**	− 0.22**	− 0.30**	− 0.27**	− 0.61**	0.38**	12.35 (5.07)					
		[-0.44, − 0.20]	[-0.59, − 0.38]	[-0.34, − 0.08]	[-0.42, − 0.18]	[-0.39, − 0.14]	[-0.68, − 0.51]	[0.26, 0.49]						
9. Emotional exhaustion		− 0.14*	− 0.34**	− 0.15*	− 0.16*	− 0.14*	− 0.35**	0.48**	0.49**	28.83 (11.19)				
		[-0.27, − 0.00]	[-0.45, − 0.21]	[-0.28, − 0.02]	[-0.29, − 0.02]	[-0.27, − 0.00]	[-0.46, − 0.22]	[0.37, 0.58]	[0.38, 0.58]					
10. Depersonalization		− 0.17*	− 0.29**	− 0.19**	− 0.16*	− 0.10	− 0.15*	0.25**	0.15*	0.53**	9.59 (6.11)			
		[-0.30, − 0.04]	[-0.41, − 0.16]	[-0.31, − 0.05]	[-0.29, − 0.03]	[-0.24, 0.03]	[-0.28, − 0.01]	[0.11, 0.37]	[0.01, 0.27]	[0.42, 0.62]				
11. Personal accomplishment		0.35**	0.34**	0.34**	0.26**	0.30**	0.27**	− 0.02	− 0.27**	− 0.20**	− 0.17*	46.41 (7.22)		
		[0.23, 0.46]	[0.21, 0.45]	[0.22, 0.46]	[0.13, 0.38]	[0.17, 0.42]	[0.14, 0.39]	[-0.15, 0.12]	[-0.39, − 0.14]	[-0.32, − 0.06]	[-0.30, − 0.04]			
12. Work Engangement		0.43**	0.41**	0.28**	0.41**	0.42**	0.33**	− 0.15*	− 0.36**	− 0.28**	− 0.23**	0.51**	4.94 (1.15)	
		[0.31, 0.53]	[0.29, 0.52]	[0.15, 0.40]	[0.29, 0.52]	[0.30, 0.52]	[0.20, 0.44]	[-0.28, − 0.02]	[-0.47, − 0.23]	[-0.40, − 0.15]	[-0.35, − 0.10]	[0.40, 0.60]		
13. Work-life balance		0.09	0.35**	0.16*	0.21**	0.15*	0.42**	− 0.38**	− 0.42**	− 0.58**	− 0.24**	0.09	0.30**	3.22 (0.92)
		[-0.05, 0.22]	[0.22, 0.46]	[0.03, 0.29]	[0.08, 0.34]	[0.02, 0.28]	[0.30, 0.52]	[-0.49, − 0.26]	[-0.52, − 0.30]	[-0.66, − 0.48]	[-0.36, − 0.11]	[-0.04, 0.23]	[0.17, 0.42]	

Scale means and standard deviations (in brackets) are presented on the diagonal of the correlation matrix. Below, the bivariate correlations between each variable pair are shown with the 95% confidence interval (values in square brackets) for each bivariate correlation. The confidence interval is a plausible range of population correlations that could have caused the sample correlation. Asterisks indicate statistically significant correlations with * indicating *p* < 0.05, and ** indicating *p* < 0.01. *N* = 212.

### Network analyses

#### Network on resilience-related factors

**Fig. 1:**
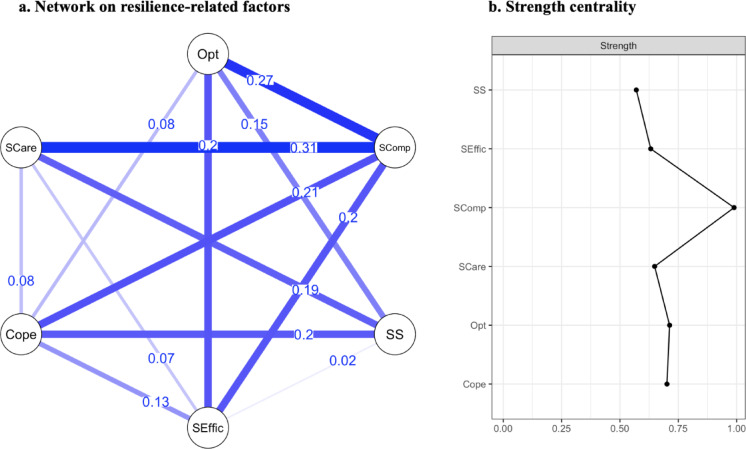
Network on resilience-related factors and strength centrality. The network of resilience-related factors (**a**) displays the interconnectedness of resilience-related factors, where each node represents a variable (Opt = optimism; Scomp = self-compassion; SS = social support; SEffic = self-efficacy; Cope = problem-focused coping; SCare = self-care). The edges between nodes represent partial correlations between those variables, controlled for the influence of other variables in the model. Blue edges indicate positive relationships; no negative relationships were detected. The width of the edges reflects the strength of the relationships. (**b**) Strength centrality of each variable (shown on the right) indicates the relative importance of each factor in the network, measured by the sum of absolute edge weights for each node.

The partial correlational network model of the resilience-related factors is presented in Fig. 1. The network consists of nodes representing resilience factors and edges representing partial correlations between these factors. Of 15 possible edges, 13 non-zero edges were included in the model, all of them being positive, indicating a resource network. The mean weight of the sample network connections was 0.14. The strongest partial correlations were found between (1) self-care and self-compassion (*r* = 0.31), (2) optimism and self-compassion (*r* = 0.27), and (3) problem-focused coping and self-compassion (*r* = 0.21). Bootstrapped confidence intervals of edge weight parameters suggested reasonable precision, although some variability was present (see supplementary material Figure S1). The edge stability analysis yielded a CS-coefficient of CS_cor=0.7_ = 0.44, indicating that the connections in the network are reasonably stable and can be interpreted. Setting the hyperparameter to γ = 0.5 for sensitivity analysis did not alter the network structure, centrality indices, or edge weights.

Centrality indices showed that self-compassion was the strongest and therefore most influential node with a value of 0.99, followed by optimism (0.71) and problem-focused coping (0.70). The strong centrality and consistent connections of self-compassion suggest that it may serve as a key leverage point within the resilience network. Accuracy analysis revealed centrality indices were interpretable with CS_cor=0.7_ = 0.36. Details of edge weights and centrality strength can be found in the supplementary material Table S1.

#### Network on resilience-related factors and indicators of stress

**Fig. 2:**
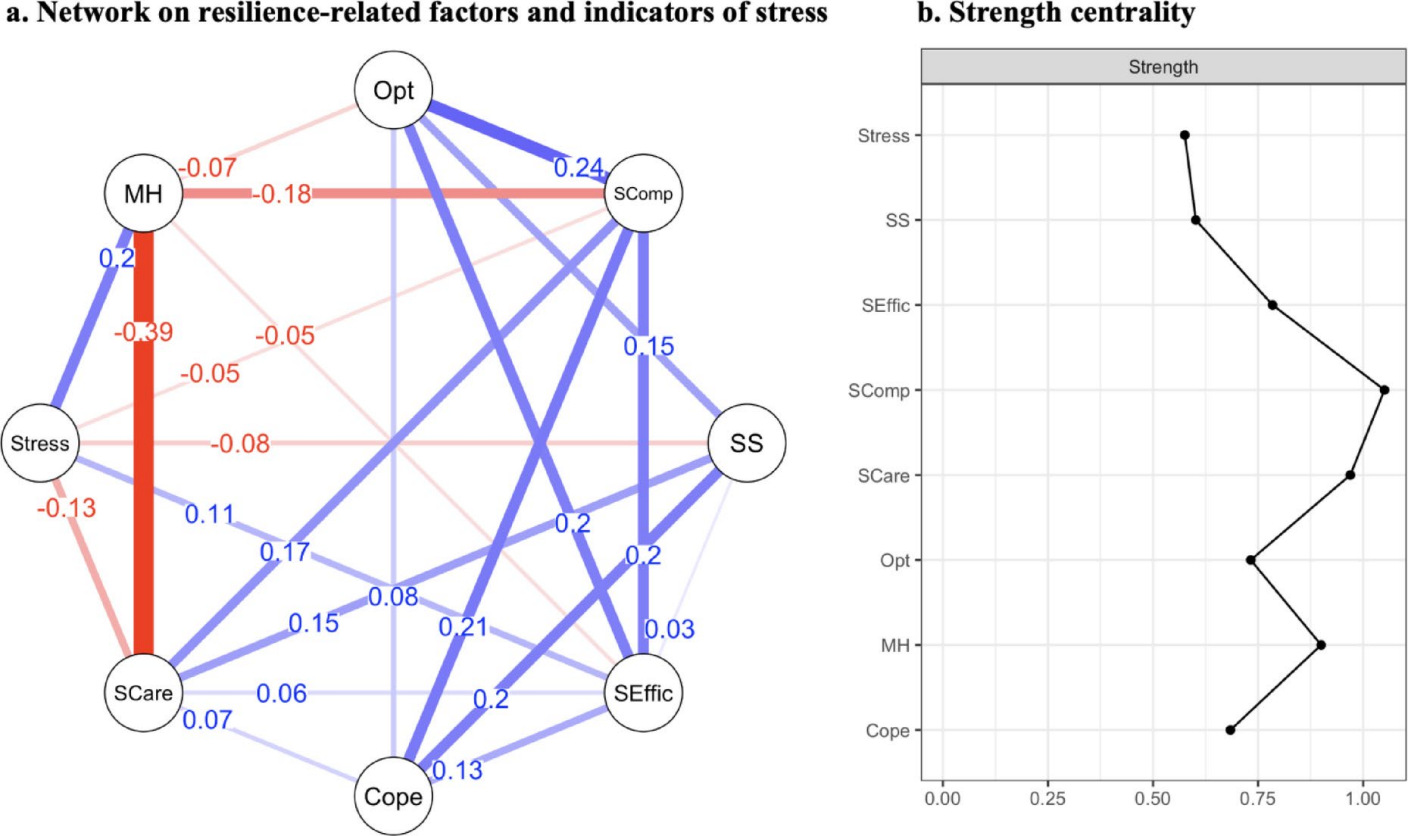
Network on resilience-related factors and indicators of stress and strength centrality. The network of resilience-related factors and indicators of stress (**a**) displays the interconnectedness of resilience-related factors with stress and mental health problems where each node represents a variable (Opt = optimism; Scomp = self-compassion; SS = social support; SEffic = self-efficacy; Cope = problem-focused coping; SCare = self-care; Stress = daily hassles; MH = mental health problems). The edges between nodes represent absolute values of partial correlations between those variables, controlled for the influence of other variables in the model. Blue edges indicate positive relationships; red lines indicate negative relationships. The width of the edges reflects the strength of the relationships. (**b**) Strength centrality of each variable (shown on the right) indicates the relative importance of each factor in the network, measured by the sum of absolute edge weights for each node.

For the second analysis, daily hassles and mental health problems were added as distinct nodes to the network on resilience-related factors, shown in Fig. 2. Of 28 possible edges, 22 survived the glasso regularization with seven of them indicating negative associations. The sample demonstrated a mean weight of 0.04, indicating overall relatively weak associations between indicators of stress and resilience factors. The strongest connections emerged between (1) mental health problems and self-care (*r* = − 0.39), optimism and self-compassion (*r* = 0.24), and (3) problem-focused coping and self-compassion (*r* = 0.21). Bootstrapped confidence intervals of edge weight parameters suggested reasonable precision, although some variability was present (see supplementary material Figure S3). Edge stability analysis revealed a CS-coefficient for edge weight accuracy of CS_cor=0.7_ = 0.59, indicating good stability of edges. Setting the hyperparameter to γ = 0.5 did not affect the overall network characteristics, supporting the robustness of our findings.

Centrality indices, again, demonstrated that self-compassion was the most central node, with a value of 1.05, followed by self-care (0.97), and mental health problems (0.90). The CS-coefficient for strength indicated centrality indices being interpretable with CS_cor=0.7_ = 0.44. Details of edge weights and centrality strength can be found in the supplementary material Table S2.

#### Network on resilience-related factors and work-related outcomes

**Fig. 3:**
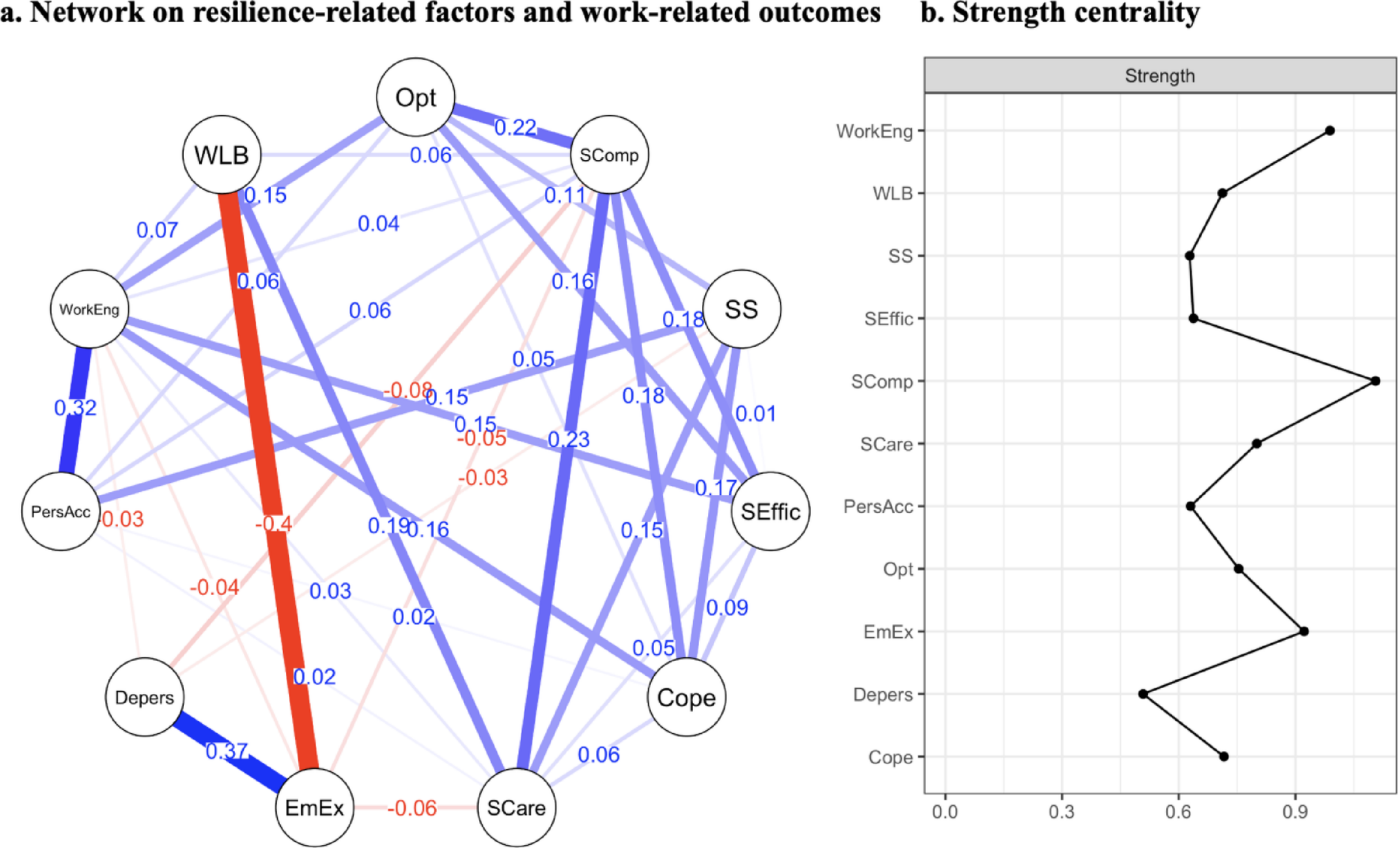
Network on resilience-related factors and work-related outcomes and strength centrality. The network of resilience-related factors and work-related outcomes (**a**) displays the interconnectedness of resilience-related factors with work-related variables, where each node represents a variable (Opt = optimism; Scomp = self-compassion; SS = social support; SEffic = self-efficacy; Cope = problem-focused coping; SCare = self-care; EmEx = emotional exhaustion (burnout); Depers = depersonalization (burnout); PersAcc = personal accomplishment (burnout); WorkEng = work engagement; WLB = work-life balance). The edges between nodes represent absolute values of partial correlations between those variables, controlled for the influence of other variables in the model. Blue edges indicate positive relationships; red lines indicate negative relationships. The width of the edges reflects the strength of the relationships. (**b**) Strength centrality of each variable (shown on the right) indicates the relative importance of each factor in the network, measured by the sum of absolute edge weights for each node.

In a third step, daily hassles and mental health problems were replaced with work-related outcomes, i.e., the facets of burnout (emotional exhaustion, depersonalization, and personal accomplishment), work-engagement and work-life balance were added to the first network on resilience-related factors (see Fig. 3). Of 55 possible edges, 35 were included, showing a small overall mean weight of 0.05. Of the included edges, 7 showed negative associations. The strongest associations were found between (1) emotional exhaustion and work-life balance (*r* = − 0.40), (2) emotional exhaustion and depersonalization (*r* = 0.37), and (3) work engagement and personal accomplishment (*r* = 0.32). Bootstrapped confidence intervals of edge weight parameters showed minor variability (see supplementary material Figure S5). The CS-coefficient indicated stable edges with an edge weight accuracy of CS_cor=0.7_ = 0.59. Setting the hyperparameter to γ = 0.5 did not alter the overall network characteristics.

Centrality indices showed that, again, self-compassion had the highest centrality strength (1.11), followed by work engagement (0.99) and emotional exhaustion (0.92). Accuracy analyses revealed a CS-coefficient of CS_cor=0.7_ = 0.44 for strength centrality, indicating moderate stability of the centrality estimates. Details of edge weights and centrality strength can be found in the supplementary material Table S3.

## Discussion

This is the first study, to our knowledge, that examined the relationships among resilience-related factors, stress, and mental health, as well as work-related outcomes in the population of HCPs in one common framework using network analysis. We were specifically interested in the role of self-compassion and self-care as potential resilience factors within this framework. Self-compassion was identified as a particularly important node in all networks, demonstrating consistently the highest centrality strength. Self-care, however, rather stands out with individual important connections to crucial factors like mental health problems and work-life balance.

The network on resilience-related factors demonstrated exclusively positive relationships, suggesting a resource network where all resilience-related factors positively reinforce each other. This indicates that enhancing central factors might lead to an overall improvement in other resilience-related factors. The most central factor in this network was self-compassion (RO1), demonstrating moderate to strong unique connections to all evidence-based resilience factors in the model, except for perceived social support. This is particularly interesting, as the previous literature has highlighted social support to be among the most important resilience factors^22,78^. While social support has been recognized for its buffering effects against the potential harmful effects of stress and in enhancing adaptive coping mechanisms, the role of self-compassion in our network points to a potentially underexplored pathway to resilience. This finding, supported by a growing body of literature suggesting that self-compassion significantly contributes to resilient outcomes^79,80^, can offer a novel perspective to the traditional focus on external social resources. Self-compassion, providing internal resources of self-regulation and a kind approach to oneself, might be complementary to social support, offering external resources in terms of validation and assistance. Both factors might contribute to resilient outcomes, impacting those via different pathways. Alternatively, it could be speculated that they might also operate along a similar pathway, as cultivating mindfulness, common humanity, and self-kindness with self-compassion reduces the reliance on external validation or support. Future longitudinal studies should examine their unique predictive value for resilient responses and their potential interplay.

To investigate potential causal effects, such studies could employ methods such as latent growth mixture modeling^81^ to examine the incremental predictive value of self-compassion for mental health trajectories following stressor exposure. Notably, a recent review synthesizing 50 primary studies on individual, social, and societal resilience factors in the context of societal challenges and crises^82^ found no investigations focusing on self-compassion, which constitutes a significant research gap. This lack of evidence underscores the need for future research to systematically evaluate the role of this factor for resilient outcomes. In addition, longitudinal mediation models or RCTs targeting self-compassion interventions could help clarify directional effects and potential mechanisms through which this factor influences resilient outcomes over time.

The strongest relationship, whilst accounting for other resilience factors, was observed between self-care and self-compassion. This indicates that higher self-compassion is associated with increased self-care among HCPs. This finding aligns with previous literature, suggesting that self-compassion may predict or promote self-care and facilitates health-promoting behaviors^37–40^. It adds to the growing evidence that targeting self-compassion could lead to favorable outcomes for both, self-compassion and self-care. This is encouraging, as self-compassion is considered a modifiable factor, and meta-analyses provide evidence that self-compassion interventions can effectively promote mental health in HCPs^83^. Studies on HCPs specifically have shown that a self-compassion intervention for healthcare communities effectively increased self-compassion and well-being, and thus reduced secondary traumatic stress and burnout^26^. Therefore, self-compassion may represent a promising and accessible target for resilience-enhancing strategies in healthcare contexts.

However, as our model included only a limited set of resilience factors, future studies could expand and explore a broader set of potentially protective variables. For instance, self-compassion has been associated with empathic concern, altruism, and a greater willingness to help others^84,85^ – qualities that are particularly relevant in helping professions. A study among nurses found that high affective empathy may increase the risk for compassion fatigue^86^, whereas mentalizing—the ability to understand one’s own and others’ mental states—was negatively associated with mental health outcomes, such as anxiety and depression in HCPs^87^. Building on this, future research could examine how mentalizing and self-compassion interact, alongside other resilience- and stress-related factors.

In our second model, the network on resilience-related factors and indicators of stress and mental health, the connections among resilience-related factors remained stable. In relation to the newly added variables, self-care particularly became important, demonstrating a strong negative connection to mental health problems (RO2). This suggests that more self-care in HCPs goes along with less mental health problems or, conversely, that more mental distress is associated with less engagement in self-care. The second strongest connection between resilience-related factors and the newly added variables was observed between self-compassion and mental health problems. This finding suggests that good mental health and a more compassionate approach to oneself appear to be intertwined. In this network, well-established resilience factors, such as optimism, social support, self-efficacy, and problem-focused coping did not show relevant associations with mental health problems when simultaneously accounting for self-care and self-compassion.

Daily hassles, measured rather objectively by the frequency of micro stressors^57^, showed low but consistent associations with various resilience-related factors after controlling for mental health problems. While prior studies have highlighted moderating effects of resilience factors on the link between stress exposure and perceived psychological distress^88^, our results show direct associations between resilience-related factors and the frequency of reported stressors. This may suggest that HCPs with higher resilience are less likely to register or report daily hassles, potentially reflecting differences in perception, attention, and/or cognitive appraisal. Strengthening resilience could therefore not only buffer stress responses but may also help reduce the perceived density of everyday stress. However, as partial correlations do not indicate a direction, it is equally possible that less struggle in everyday life (i.e., lower frequencies of daily hassles) makes it easier to engage in self-care or maintain self-compassion or, reversely, that with experiencing more daily hassles the capacity to engage in resilience strategies becomes more challenging, resulting in decreased resources.

The connection between daily hassles and self-efficacy, however, was found to be positive. This aligns with previous stress research suggesting that self-efficacy can be a resource regarding stress-vulnerability, as individuals with higher self-efficacy are more likely to view challenges as manageable rather than threatening^89,90^. In this sense, self-efficacy might only become “visible” at a certain initial level of challenge or stress. Recent approaches based on Lazarus’ stress theory, also link self-efficacy with stressors, suggesting a sequential progression of appraising a stimulus as positive, irrelevant, or threatening and then evaluating someone’s resources^91^. If these resources are insufficient, perceived stress emerges, whereas evaluating resources as sufficient can be understood as being self-effective. The positive link between self-efficacy and problem-focused coping in our sample points into the direction that more self-efficacy goes along with more adaptive coping, or vice versa, being aware of one’s own coping strategies might boost self-efficacy, which is supported by previous studies^92^. The lack of a direct link between daily hassles and problem-focused coping, however, may support Lazarus’ claim of first evaluating one’s own resources before engaging in coping.

In our third model, i.e., the network on resilience-related factors and work-related outcomes, the burnout facets shared overall little connections with resilience-related factors, whereas work-engagement turned out to be closely interrelated (RO3). While previous literature found significant associations between all components of burnout and self-compassion^93^, those connections were rather small in our network. This is likely due to the different variables we accounted for, and calls for further research. While depersonalization and emotional exhaustion, the stress-related facets of burnout, demonstrated a strong connection with each other, no link was found to the third facet of burnout, personal accomplishment. Personal accomplishment, in turn, showed a strong association with work-engagement and small to medium connections to most resilience-related factors. This ties in with criticism of the MBI, stating that the scale personal accomplishment rather reflects work engagement than burnout^94^. The small to medium connections between work-engagement and almost all resilience factors, however, can be well embedded into the findings of previous studies, indicating that both, job and personal demands and resources contribute to employees’ well-being at work, with bidirectional links between personal resources such as self-esteem, self-efficacy, and optimism, and increased work engagement^95^. This could indicate that improving work engagement and reducing burnout require distinct interventions, even though the concepts are related^96^.

The absence of a link between emotional exhaustion and problem-focused coping could go along with the process model of burnout^97^. In this model, emotional exhaustion is a reaction to chronic occupational stressors, with disengagement from clients (i.e., depersonalization) being a key coping strategy. This is in line with the strong connection between emotional exhaustion and depersonalization in our data. Furthermore, the very strong link between emotional exhaustion and work-life balance indicates that, as emotional exhaustion increases, the ability to maintain a healthy work-life balance diminishes or, vice versa, that a suboptimal work-life balance leads to emotional exhaustion. This imbalance, compounded by the lack of healthy coping mechanisms, makes individuals vulnerable to experiencing burnout^98^. In this context, self-care, which is positively associated with work-life balance, may play a crucial role in mitigating these effects and preventing burnout.

To conclude, other studies have shown that key determinants of burnout in HCPs include workload and workplace relationships, while protective factors include role clarity, professional autonomy, a sense of fair treatment, and access to regular clinical supervision^99^. Further, the literature suggests that having adequate staff, good administrative support for nursing care, and good relations between doctors and nurses were linked to positive outcomes for both nurses and patients^8^. The workplace therefore seems to play a critical role in burnout, job satisfaction, and patient satisfaction, while in our data, individual resilience factors showed rather weak associations with burnout. Thus, while not subject of our analyses, interventions aiming to promote resilience may start with modifying working conditions, as studies have shown that heavy workloads prevent HCPs from engaging in self-care^100^. Further, studies found that HCPs perceive a necessity for authorization from their environment when it comes to engaging in self-compassion, and that they feel encouraged and supported when working with self-compassionate colleagues^100,101^. Therefore, one idea could be to intervene at the organizational level, with embedding a self-compassion-cultivating mindset at the workplace, rather than intervening solely at the individual level.

Our findings on the interrelations between resilience-related factors, stress, mental health problems, and work-related outcomes provide valuable insights into demands and resources in HCPs, while also revealing the complexity of the investigated relationships. Our results not only relate well to existing literature but also revealed new insights by considering all relationships simultaneously, while accounting for each other through network modeling. This approach not only demonstrated that previously established individual connections still hold true when examined together but also revealed central factors playing a key role in the set of variables examined.

Some limitations have, however, to be taken into consideration when interpreting the results of the present study. First, as this analysis was conducted retrospectively using data collected for an RCT, there was no a priori power analysis for network modeling. Therefore, we had a rather small sample, which may have led to underpowered results. To address this limitation, we followed recommendations on how to deal with skewed ordinal categorical data from small samples^72^ and estimated the accuracy of our results according to recent recommendations^73^. Additionally, our sample was not representative for the German HCP population, which limits the generalizability of our findings. Replications with larger and representative samples are needed for robust conclusions. As we initially collected data for testing an online resilience training program, we focused on healthy individuals for this training, excluding participants with high levels of mental burden (e.g., reporting suicidal ideation, receiving psychotherapy or psychiatric treatment). This may not reflect the average HCP population, as HCPs were found to be at heightened risk for experiencing mental health problems^3–5,102^.

Second, we want to highlight that the stability of strength centrality measures, as indicated by the CS-coefficients, must be interpreted with caution. While all our results met the criteria for interpretability by staying above the minimum threshold of > 0.25, the first two networks remained below the ideal threshold of > 0.5. However, this may rather reflect a general feature of ordinal categorical data which are known to have problematic centrality results^72^. Having this in mind, our results might be viewed as still acceptable. Third, the cross-sectional design limits the ability to draw conclusions about causality and directionality. Longitudinal studies are needed to shed light on the directionality of associations and interactions between the factors over time. While our aim was not mainly to validate the concept of resilience itself, but rather to look at the interactions of selected resilience-related factors at a given time point, it has to be noted that resilience is best assessed using a longitudinal approach to capture its dynamic nature^103^. Lastly, as mentioned above, we did not claim to paint an all-encompassing picture of resilience. Therefore, it is important to acknowledge that our findings may be confounded by other unmeasured variables not considered in our models.

Our findings provide a valuable foundation for future longitudinal studies to investigate the role of self-care and self-compassion in maintaining and regaining mental health under stress, which could inform the development of resilience-promoting interventions for HCPs. Self-compassion appeared to be the strongest node in all models, showing throughout consistent connections to most variables. The stability and strength of self-compassion across all networks, raises the question whether it may potentially, through its underlying healthy relationship with oneself, serve as a fundamental mechanism for other resilience factors, possibly be acting as an “engine”. The relevance of self-care was particularly evident in relation to mental health problems and work-life balance, suggesting that self-care may not only be closely related to mental health but may also play a role in sustaining a good work-life balance. The limited interconnectedness of stress-related burnout facets to resilience-related factors, with the only strong negative link to work-life, balance indicates that burnout seems more connected to external factors such as the balance between work and personal life or other variables not examined, like the work environment itself. Future studies will be needed to answer the open questions raised by our findings, clarify potentially causal influences, and investigate potential benefits of organizational interventions cultivating a self-compassionate mindset at the workplace.

## Electronic supplementary material

Below is the link to the electronic supplementary material.

Supplementary Material 1

## Data Availability

Data are available from the corresponding author upon reasonable request.
