# Author Correction: The role of self-care and self-compassion in networks of resilience and stress among healthcare professionals

**DOI:** 10.1038/s41598-025-33287-x

**Published:** 2025-12-30

**Authors:** Carolina Pank, Lisa von Boros, Klaus Lieb, Nina Dalkner, Sebastian Egger-Lampl, Dirk Lehr, Sarah K. Schäfer, Oliver Tüscher, Michèle Wessa

**Affiliations:** 1 Leibniz Institute for Resilience Research, Mainz, Germany; 2 Department of Psychiatry and Psychotherapy, University Medical Center of Johannes Gutenberg University Mainz, Mainz, Germany; 3 Department of Psychiatry and Psychotherapeutic Medicine, Medical University Graz, Graz, Austria; 4 Mindconsole GmbH, Graz, Austria; 5 Institute for Sustainability Education and Psychology, Department of Health Psychology and Applied Biological Psychology, Leuphana University, Lüneburg, Germany; 6 Department of Clinical Psychology, Psychotherapy and Psychodiagnostics, Technical University Braunschweig, Braunschweig, Germany; 7 Department of Psychiatry, Psychotherapy and Psychosomatic Medicine, Martin-Luther University Halle-Wittenberg, Halle, Germany; 8 Central Institute of Mental Health, Mannheim, Germany; 9 DKFZ Hector Cancer Institute at the University Medical Center Mannheim, Heidelberg, Germany; 10 German Cancer Research Center (DKFZ) Heidelberg, Division C160, Heidelberg, Germany

Correction to: *Scientific Reports* 10.1038/s41598-025-01111-1, published online 27 May 2025

The original version of this article contained errors in the reported numerical values from the third network model.

As a result, in the Abstract, where:

“Cross-sectional data from HCPs in Germany were collected from June-July 2023. Analyses of 212 HCPs (age 41.63 [21–68] years; 81.60% women) revealed self-compassion as the most important factor across all networks, while the importance of self-care showed through individual connections to crucial factors like mental health problems and work-life balance.”

now reads:

“Cross-sectional data from HCPs in Germany were collected in April 2023. Analyses of 212 HCPs (age 41.63 [21–68] years; 81.60% women) revealed self-compassion as the most important factor across all networks, while the importance of self-care showed through individual connections to crucial factors like mental health problems and work-life balance.”

In addition, under the Results section, subheading ‘Network on resilience-related factors and work-related outcomes’, where:

“Of 78 possible edges, 48 were included, showing a small overall mean weight of 0.03. Of the included edges, 16 showed negative associations. The strongest associations were found between (1) emotional exhaustion and depersonalization (*r* = 0.35), (2) self-care and mental health problems (*r* = − 0.34), and (3) emotional exhaustion and work-life balance (*r* = − 0.34). Bootstrapped confidence intervals of edge weight parameters showed minor variability (see supplementary material Figure S5). The CS-coefficient indicated stable edges with an edge weight accuracy of CS_cor=0.7_ = 0.59. Setting the hyperparameter to γ = 0.5 did not alter the overall network characteristics.”

now reads:

“Of 55 possible edges, 35 were included, showing a small overall mean weight of 0.05. Of the included edges, 7 showed negative associations. The strongest associations were found between (1) emotional exhaustion and work-life balance (*r* = − 0.40), (2) emotional exhaustion and depersonalization (*r* = 0.37), and (3) work engagement and personal accomplishment (*r* = 0.32). Bootstrapped confidence intervals of edge weight parameters showed minor variability (see supplementary material Figure S5). The CS-coefficient indicated stable edges with an edge weight accuracy of CS_cor=0.7_ = 0.59. Setting the hyperparameter to γ = 0.5 did not alter the overall network characteristics.”

And where:

“Centrality indices showed that, again, self-compassion had the highest centrality strength (1.11), followed by work engagement (0.99) and emotional exhaustion (0.92). Accuracy analyses revealed a CS-coefficient of CS_cor=0.7_ = 0.52 for strength centrality, indicating that the centrality indices are stable, and the differences can be reliably interpreted. Details of edge weights and centrality strength can be found in the supplementary material Table S3.”

now reads:

“Centrality indices showed that, again, self-compassion had the highest centrality strength (1.11), followed by work engagement (0.99) and emotional exhaustion (0.92). Accuracy analyses revealed a CS-coefficient of CS_cor=0.7_ = 0.44 for strength centrality, indicating moderate stability of the centrality estimates. Details of edge weights and centrality strength can be found in the supplementary material Table S3.”

The original version of this Article has been corrected.

