# Supplementary material for: The role of self-care and self-compassion in networks of resilience and stress among healthcare professionals
Source: Sci Rep. 2025 May 27;15:18545. doi: 10.1038/s41598-025-01111-1 (PMC12116801; doi:10.1038/s41598-025-01111-1)
Supplement: Supplementary file 1 — Supplementary Material 1 [file 41598_2025_1111_MOESM1_ESM.pdf]

SUPPLEMENTARY MATERIAL

The Role of Self-Care and Self-Compassion in Networks of Resilience and Stress Among Healthcare Professionals

Authors: Carolina Pank[1], Lisa von Boros[1], Klaus Lieb[1,2], Nina Dalkner[3], Sebastian Egger-Lampl[4], Dirk Lehr[5], Sarah K. Schäfer[1,6,§], Oliver Tüscher[1,2,7,§], Michèle Wessa[1,8,9 §,*]

[1] Leibniz Institute for Resilience Research, Mainz, Germany
[2] Department of Psychiatry and Psychotherapy, University Medical Center of Johannes Gutenberg University Mainz, Mainz, Germany
[3] Department of Psychiatry and Psychotherapeutic Medicine, Medical University Graz, Graz, Austria
[4] Mindconsole GmbH, Graz, Austria
[5] Institute for Sustainability Education and Psychology, Department of Health Psychology and Applied Biological Psychology, Leuphana University, Lüneburg, Germany
[6] Clinical Psychology, Psychotherapy and Psychodiagnostics, Technical University Braunschweig, Braunschweig, Germany
[7] Department of Psychiatry, Psychotherapy and Psychosomatic Medicine, University Medicine Halle, Martin-Luther University Halle-Wittenberg, Halle, Germany
[8] German Cancer Research Center (DKFZ) Heidelberg, Division C160, Germany
[9] Central Institute of Mental Health, Mannheim, Germany

*Correspondence concerning this article should be addressed to Michèle Wessa, Leibniz Institute for Resilience Research, Wallstraße 7, 55122 Mainz,
Email: Michèle.Wessa@lir-mainz.de

[§] shared last authors

Table of contents

S1 Bootstrapped confidence intervals of edge weight parameters and centrality stability.. 3

Figure S1 Bootstrapped confidence intervals of edge weight parameters for the network on resilience-related factors. 3

Figure S2 Centrality stability for the network on resilience related factors 4

Figure S3 Bootstrapped confidence intervals of edge weight parameters for the network on resilience-related factors and indicators of stress. 5

Figure S4 Centrality stability for the network on resilience related factors and indicators of stress. 6

Figure S5 Bootstrapped confidence intervals of edge weight parameters for the network on resilience-related factors and work-related outcomes. 7

Figure S6 Centrality stability for the network on resilience related factors and work-related outcomes. 8

S2 Edges weights and centrality strength 8

Table S1. Edge weights and centrality strength of the network on resilience-related factors 8

Table S2. Edge weights and centrality strength of the network on resilience-related factors and indicators of stress 9

9

Table S3. Edge weights and centrality strength of the network on resilience-related factors and work-related outcomes 9

S3 Details on analyses in R 10

S1 Bootstrapped confidence intervals of edge weight parameters and centrality stability

Figure S1. Bootstrapped confidence intervals of edge weight parameters for the network on resilience-related factors

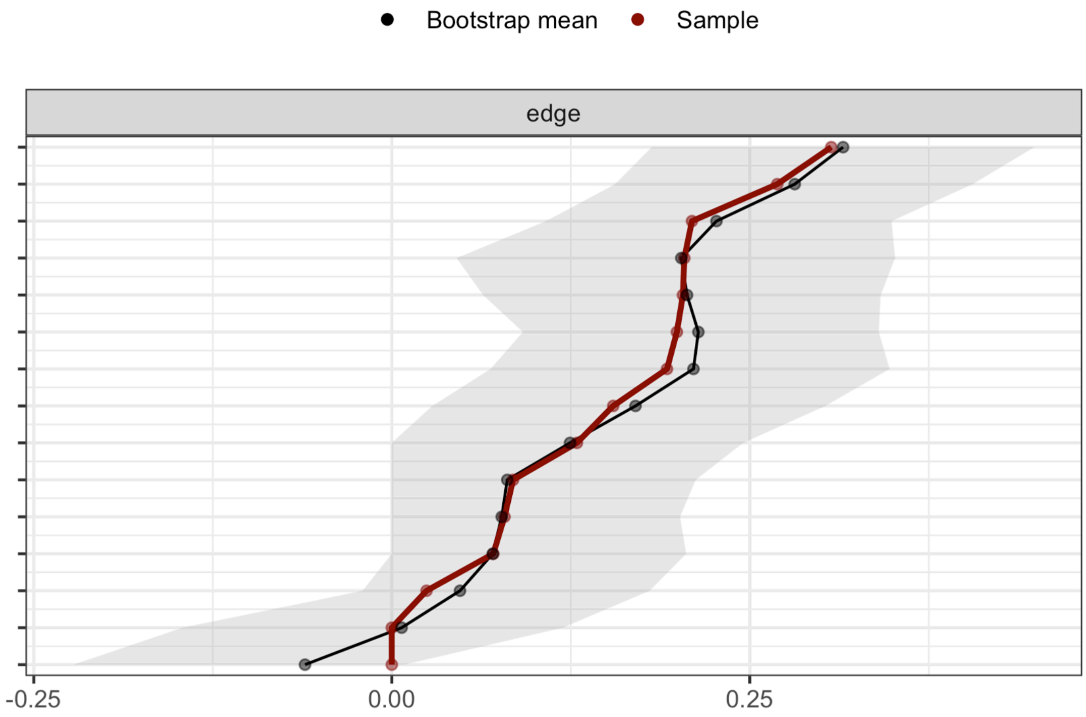

Figure S1 Bootstrapped confidence intervals of edge weight parameters for the network on resilience-related factors. The red line depicts the sample edge weights, while the black line with represents the mean edge weights from the bootstrap samples. The gray bar depicts the bootstrapped 95% confidence intervals around the edge weights.

Figure S2. Centrality stability for the network on resilience-related factors

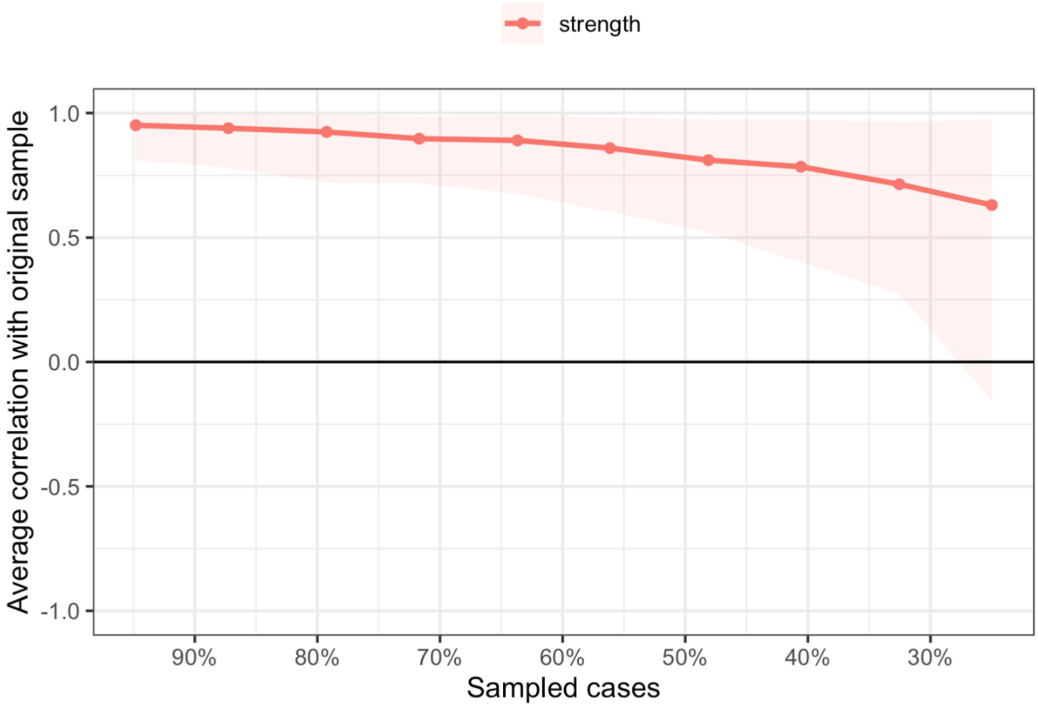

Figure S2 Centrality stability for the network on resilience related factors. This figure displays the centrality stability for the network on resilience-related factors using the correlation stability (CS) coefficient. The x-axis represents the proportion of sampled cases, and the y-axis shows the average correlation of centrality estimates with the original sample. The red line represents the average correlation between strength centrality of network sampled with persons dropped and the original sample. The red line indicates the mean and the shaded area indicate the range from the 2.5th quantile to the 97.5th quantile.

Figure S3. Bootstrapped confidence intervals of edge weight parameters for the network on resilience-related factors and indicators of stress

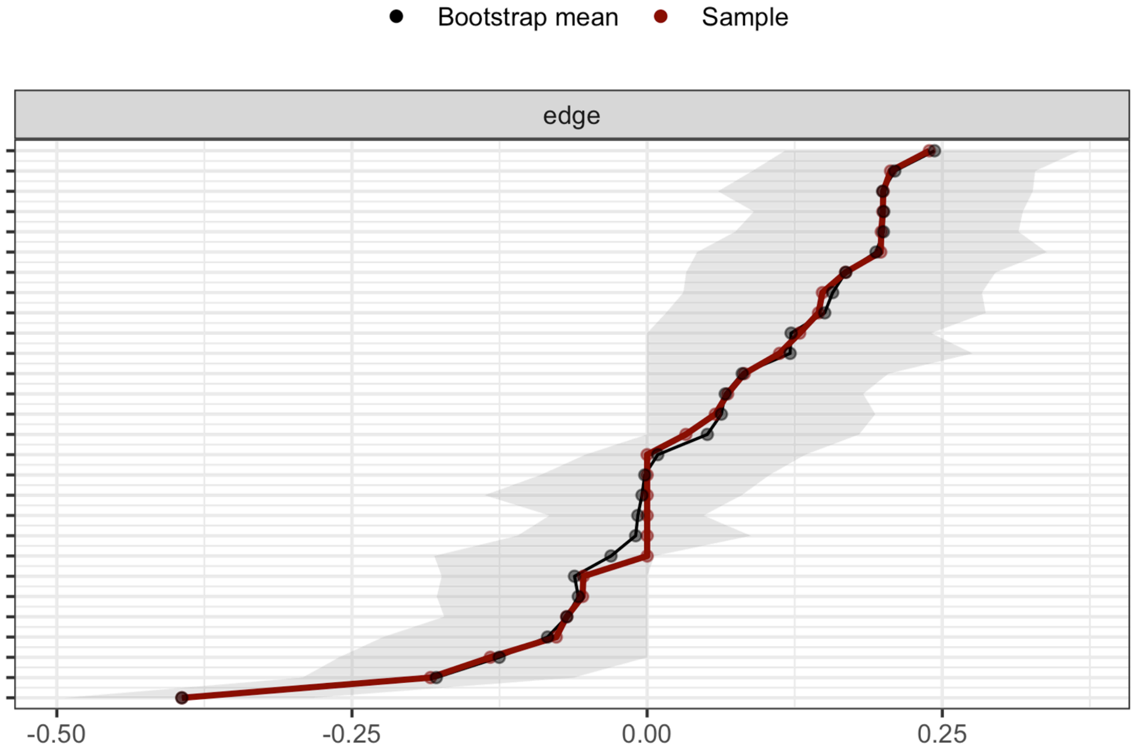

Figure S3 Bootstrapped confidence intervals of edge weight parameters for the network on resilience-related factors and indicators of stress. The red line depicts the sample edge weights, while the black line with represents the mean edge weights from the bootstrap samples. The gray bar depicts the bootstrapped 95% confidence intervals around the edge weights.

Figure S4. Centrality stability for the network on resilience-related factors and indicators of stress

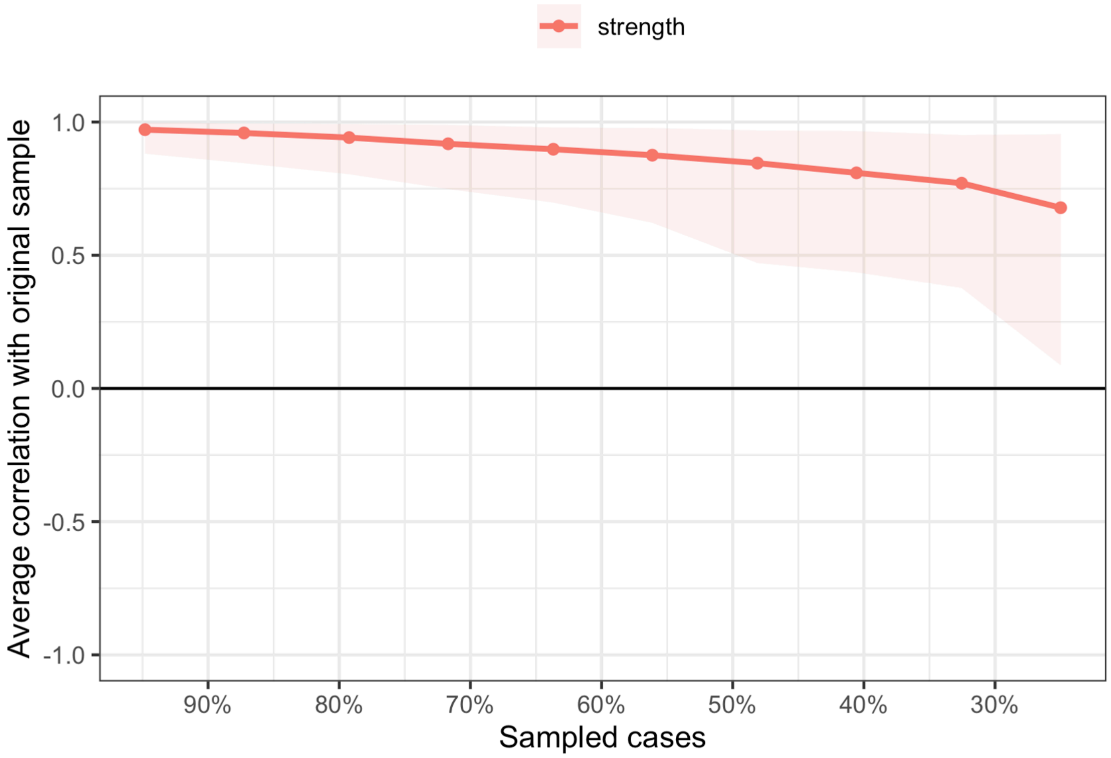

Figure S4 Centrality stability for the network on resilience related factors and indicators of stress. This figure displays the centrality stability for the network on resilience-related factors and indicators of stress using the correlation stability (CS) coefficient. The x-axis represents the proportion of sampled cases, and the y-axis shows the average correlation of centrality estimates with the original sample. The red line represents the average correlation between strength centrality of network sampled with persons dropped and the original sample. The red line indicates the mean and the shaded area indicate the range from the 2.5th quantile to the 97.5th quantile.

Figure S5. Bootstrapped confidence intervals of edge weight parameters for the network on resilience-related factors and work-related outcomes

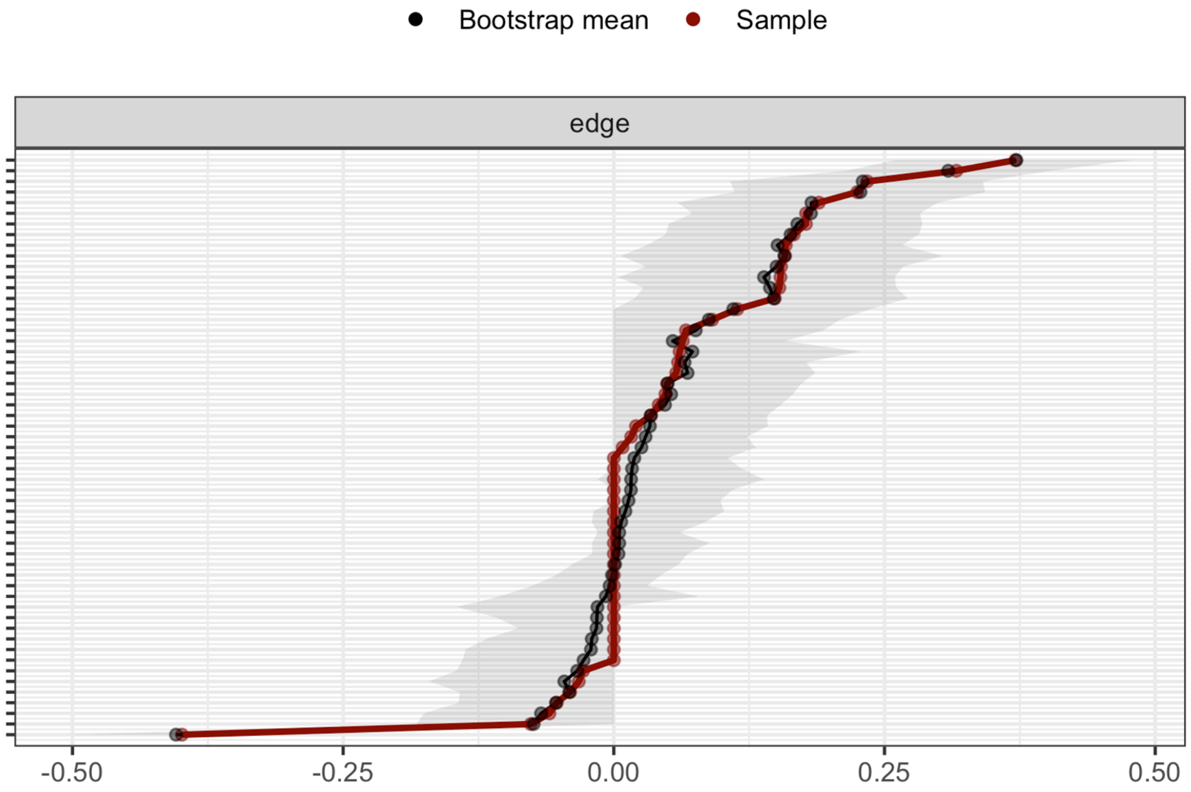

Figure S5 Bootstrapped confidence intervals of edge weight parameters for the network on resilience-related factors and work-related outcomes. The red line depicts the sample edge weights, while the black line with represents the mean edge weights from the bootstrap samples. The gray bar depicts the bootstrapped 95% confidence intervals around the edge weights.

Figure S6. Centrality stability for the network on resilience-related factors and work-related outcomes

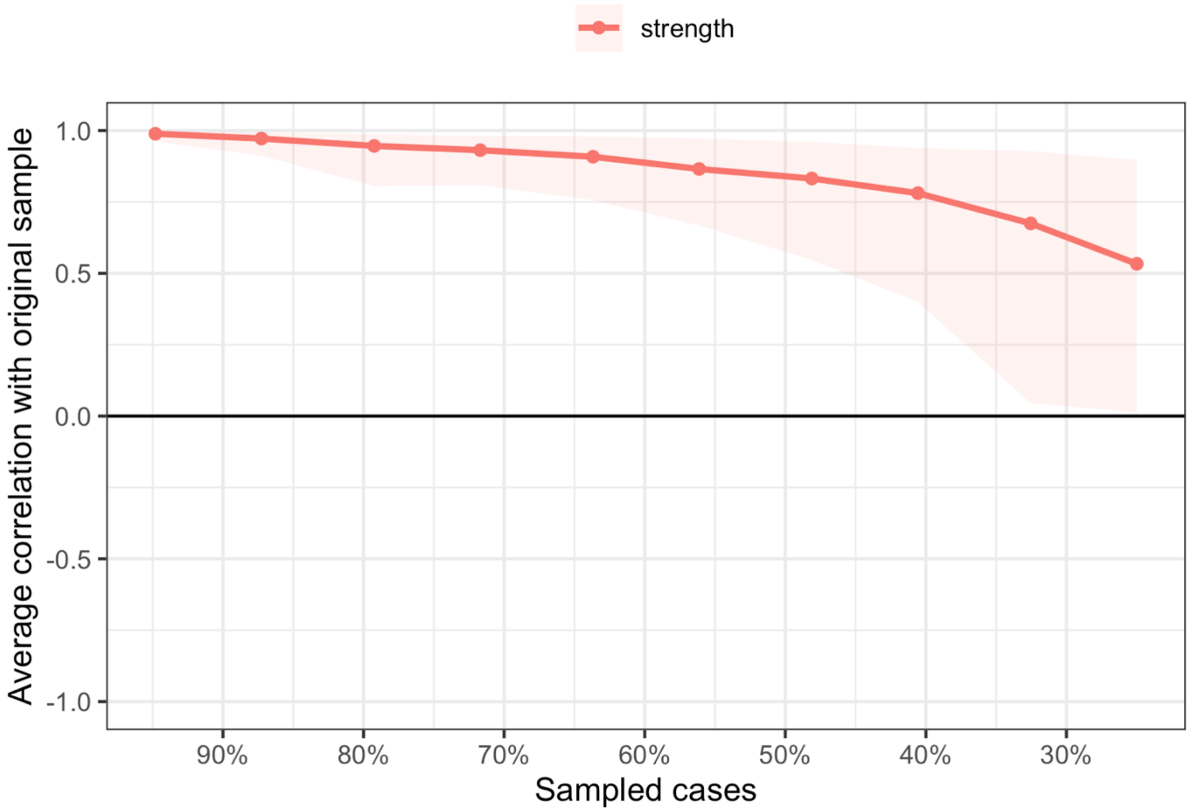

Figure S6 Centrality stability for the network on resilience related factors and work-related outcomes. This figure displays the centrality stability for the network on resilience-related factors and work-related outcomes using the correlation stability (CS) coefficient. The x-axis represents the proportion of sampled cases, and the y-axis shows the average correlation of centrality estimates with the original sample. The red line represents the average correlation between strength centrality of network sampled with persons dropped and the original sample. The red line indicates the mean and the shaded area indicate the range from the 2.5th quantile to the 97.5th quantile

S2 Edges weights and centrality strength

Table S1. Edge weights and centrality strength of the network on resilience-related factors

	Opt	SComp	SS	SEffic	Cope	SCare
Opt	**0.712**					
SComp	0.269	**0.989**				
SS	0.155	0	**0.57**			
SEffic	0.204	0.203	0.024	**0.632**		
Cope	0.085	0.21	0.199	0.129	**0.701**	
SCare	0	0.307	0.192	0.07	0.079	**0.649**

Note. The diagonal values represent the strength centrality of each node (i.e., the sum of absolute edge weights connected to that node), while the off-diagonal values indicate the edge weights between pairs of variables. Higher strength centrality values indicate nodes with stronger overall connections to other variables in the network. Opt = optimism; Scomp = self-compassion; SS = social support; SEffic = self-efficacy; Cope = problem-focused coping; SCare = self-care

Table S2. Edge weights and centrality strength of the network on resilience-related factors and indicators of stress

	Opt	SComp	SS	SEffic	Cope	SCare	Stress	MH
Opt	**0.733**							
SComp	0.239	**1.051**						
SS	0.145	0	**0.602**					
SEffic	0.198	0.2	0.033	**0.784**				
Cope	0.082	0.206	0.198	0.129	**0.684**			
SCare	0	0.168	0.148	0.058	0.068	**0.969**		
Stress	0	-0.055	-0.077	0.112	0	-0.133	**0.576**	
MH	-0.068	-0.184	0	-0.055	0	-0.394	0.2	**0.9**

Note. The diagonal values represent the strength centrality of each node (i.e., the sum of absolute edge weights connected to that node), while the off-diagonal values indicate the edge weights between pairs of variables. Higher strength centrality values indicate nodes with stronger overall connections to other variables in the network. Opt = optimism; Scomp = self-compassion; SS = social support; SEffic = self-efficacy; Cope = problem-focused coping; SCare = self-care; Stress = daily hassles; MH = mental health problems.

Table S3. Edge weights and centrality strength of the network on resilience-related factors and work-related outcomes

	Opt	SComp	SS	SEffic	Cope	SCare	EmEx	Depers	PersAcc	WorkEng	WLB
Opt	**0.754**										
SComp	0.224	**1.106**									
SS	0.114	0	**0.628**								
SEffic	0.158	0.177	0.008	**0.638**							
Cope	0.048	0.178	0.166	0.091	**0.716**						
SCare	0	0.234	0.155	0.049	0.059	**0.8**					
EmEx	0	-0.053	0	0	0	-0.059	**0.922**				
Depers	0	-0.077	-0.032	0	0	0	0.371	**0.508**			
PersAcc	0.061	0.064	0.152	0	0.016	0.02	0	0	**0.63**		
WorkEng	0.149	0.041	0	0.154	0.159	0.034	-0.04	-0.028	0.316	**0.989**	
WLB	0	0.058	0	0	0	0.189	-0.399	0	0	0.067	**0.712**

Note. The diagonal values represent the strength centrality of each node (i.e., the sum of absolute edge weights connected to that node), while the off-diagonal values indicate the edge weights between pairs of variables. Higher strength centrality values indicate nodes with stronger overall connections to other variables in the network. Opt = optimism; Scomp = self-compassion; SS = social support; SEffic = self-efficacy; Cope = problem-focused coping; SCare = self-care; EmEx = emotional exhaustion (burnout); Depers = depersonalization (burnout); PersAcc = personal accomplishment (burnout); WorkEng = work engagement; WLB = work-life-balance

S3 Details on analyses in R

```
# Code for network modeling in R version 4.2.3

# Load packages
library('bootnet')
library('qgraph')
library('polycor')

# Exemplary network model: Network on resilience-related factors
# Subdataset Model 1
resilience_network <- subset(HCP_resilir, select = c("Optionly",
"SelfCompassion", "SocialSupport", "SelfEfficacy", "ProbFocCope",
"SelfCare"))

# Network model using EBICglasso
Model1 <- estimateNetwork(
  resilience_network,
  default = "EBICglasso",
  corMethod = "cor_auto",
  tuning = 0.25)

#Plot network including predictability in colorblind version
edge_weights <- Model1$graph
edge_colors <- ifelse(edge_weights > 0, "blue", "red")
custom_labels <- c("Opt", "SComp", "SS", "SEffic", "Cope", "SCare")
qgraph(Model1$graph,
       layout = "circle",
       labels = custom_labels,
       title = "HCP Resilience Network",
       label.cex = 1.0,
       vsize = 8,
       color = "white",
       edge.color = edge_colors,
       edge.width = 1.0,
       edge.labels = TRUE,
       edge.label.cex = 1.2,
       edge.label.color = edge_colors,
       edge.label.position = 0.2,
       edge.label.bg = "white",
       title = "etwork on resilience-related factors"
)

# extract the weighted adjacency matrix
getWmat(Model1)

#centrality
centralityPlot(Model1)

# edge weight accuracy
boot1 <- bootnet(Model1, nCores = 8, nBoots = 2500, type =
'nonparametric')

# bootnet plot visualized
plot(boot1, labels = FALSE, order = "sample")

# bootnet Results
```

```
print(boot1)

# Table of all statistics from original sample
boot1$sampleTable
```

# Centrality stability

```
boot2 <- bootnet(Model1, nCores = 8, nBoots = 2500, type = 'case')

# visualizing
plot(boot2)

# values
corStability(boot2)
```
